# Exciton Manipulation via Dielectric Environment Engineering in 2D Semiconductors

**DOI:** 10.1021/acsaom.5c00105

**Published:** 2025-05-20

**Authors:** Raziel Itzhak, Nathan Suleymanov, Boris Minkovich, Liana Kartvelishvili, Vladislav Kostianovski, Roman Korobko, Alex Hayat, Ilya Goykhman

**Affiliations:** † Department of Electrical and Computer Engineering, Technion, Haifa 32000, Israel; ‡ Institute of Applied Physics, The Faculty of Science and The Center for Nanoscience and Nanotechnology, 26742 The Hebrew University of Jerusalem, Jerusalem 91904, Israel

**Keywords:** 2D materials, monolayer TMDs, excitons, dielectric environment, binding energy, photoluminescence, nano-optoelectronics

## Abstract

Two-dimensional (2D) semiconductors are promising for photonic applications due to their exceptional optoelectronic properties, including large exciton binding energy, strong spin–orbit coupling, and potential integration with the standard complementary silicon-oxide-semiconductor (CMOS) technology. The dielectric environment can significantly affect the photoluminescence (PL) spectra of transition metal dichalcogenide (TMD) monolayers by modulating excitonic properties such as optical transitions and binding energies. Specifically, substrates with higher dielectric permittivity reduce exciton binding energy and the quasiparticle bandgap. Doping and the charge carrier concentration can further modify the emitted spectra by affecting the PL excitonic content. Increased doping can enhance trion formation and bandgap renormalization phenomena, leading to PL spectral shifts that depend on the semiconductor type. This study systematically investigates the substrate-induced dielectric screening, doping, and trapped charges in CVD-grown n-type 1L-WS_2_ and p-type 1L-WSe_2_ transferred onto CMOS-relevant SiO_2_ and HfO_2_ dielectrics. Our results show that p-type 1L-WSe_2_ exhibits higher PL intensity and red-shifted trion emission on HfO_2_, whereas n-type 1L-WS_2_ shows a blue-shifted, lower-intensity PL for a similar dielectric environment. The difference arises from the interplay of the semiconductor type, doping, dielectric screening, and charge carrier concentration. We demonstrate that suspending the monolayers at the nanoscale enhances PL by reducing nonradiative recombination, enabling controlled micro-PL patterning and the formation of localized emission hot spots. Our results provide valuable insights for the development of next-generation CMOS-compatible optoelectronic devices.

## Graphical abstract

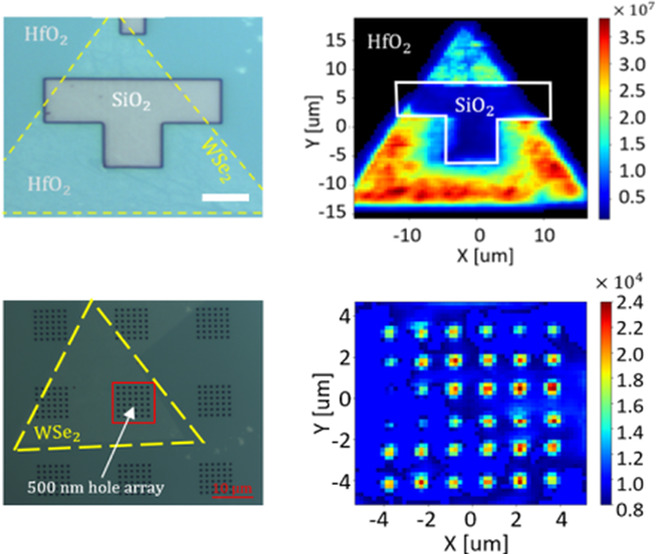

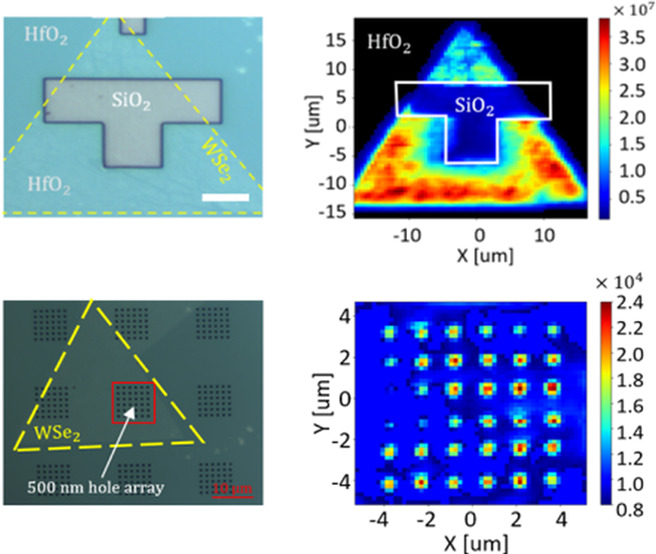

## Introduction

Optically active 2D semiconductors have recently attracted significant interest in the development of novel, atomically thin optoelectronics devices,^1^ including photodetectors,^1–3^ optical modulators,^4–8^ and light-emitting diodes.^9–11^ One of the most studied families of 2D semiconductors are TMDs, which have the formula of MX_2_, where M denotes a transition metal atom (e.g., Mo and W) and X denotes a chalcogen atom (e.g., S and Se).^12–15^ Single-layer (1L) TMDs exhibit a unique combination of large exciton binding energy on the order of hundreds of meV,^16–18^ strong spin–orbit coupling,^19,20^ and optically addressable valley degree of freedom.^21–23^ These properties are from the presence of heavy metal ions in TMD crystals,^24^ reduced Coulomb screening in two dimensions,^16^ and lack of inversion symmetry in TMD monolayers.^23^ Various 2D TMDs have a direct energy bandgap in the visible (e.g., 1L-MoS_2_ and 1L-WS_2_)^22,25–27^ and near-infrared (e.g., 1L-WSe_2_, and InSe)^28,29^ wavelengths, allowing for radiative recombination and light emission with distinct excitonic features,^25^ tunable optical bandgap,^30^ and rich excitonic content.^31,32^

In recent years, intensive studies have been conducted on exciton manipulation in optically active TMDs using different dielectric environments and charge carrier concentrations in 2D layers.^17,33–37^ Figure 1 shows the schematic energy band diagram of an optically active semiconducting material with a direct energy bandgap *E*_g_ = *E*_C_ – *E*_V_, where *E*_C_ is the minimum of the conduction band and *E*_V_ is the maximum of the valence band. The optical transition *E*_*x*_^0^ depicts the excitonic properties of the system, where *X* ^0^ denotes the neutral exciton with a binding energy of *E*_b_. Due to the atomic layer thickness, the excitonic properties in 2D TMDs, including *E*_*x*_^0^ and *E*_b_, can be modulated by the dielectric permittivity ε_r_ of the surrounding materials like a substrate^35,38–40^ or top encapsulation layer.^41–44^ Specifically, using a substrate with a larger ε_r_ tends to weaken Coulomb interactions and decrease the exciton binding energy to *E*_b_′ and the energy bandgap to *E*_g_′ by Δ*E*_b_ and Δ*E*_g_, respectively (Figure 1a). This leads to a typical PL redshift of Δ*E*_*x*_^0^ when |Δ*E*_g_| > |Δ*E*_b_|, resulting in light emission at longer wavelengths with a reduced optical bandgap.^45,46^ Still, using a substrate with ultrahigh ε_r_ ∼ 1000 can lead to PL blueshift (i.e., |Δ*E*_g_| < |Δ*E*_b_|) due to the breaking of the static dielectric screening approximation of the exciton Coulomb interaction.^47^

**Figure 1:**
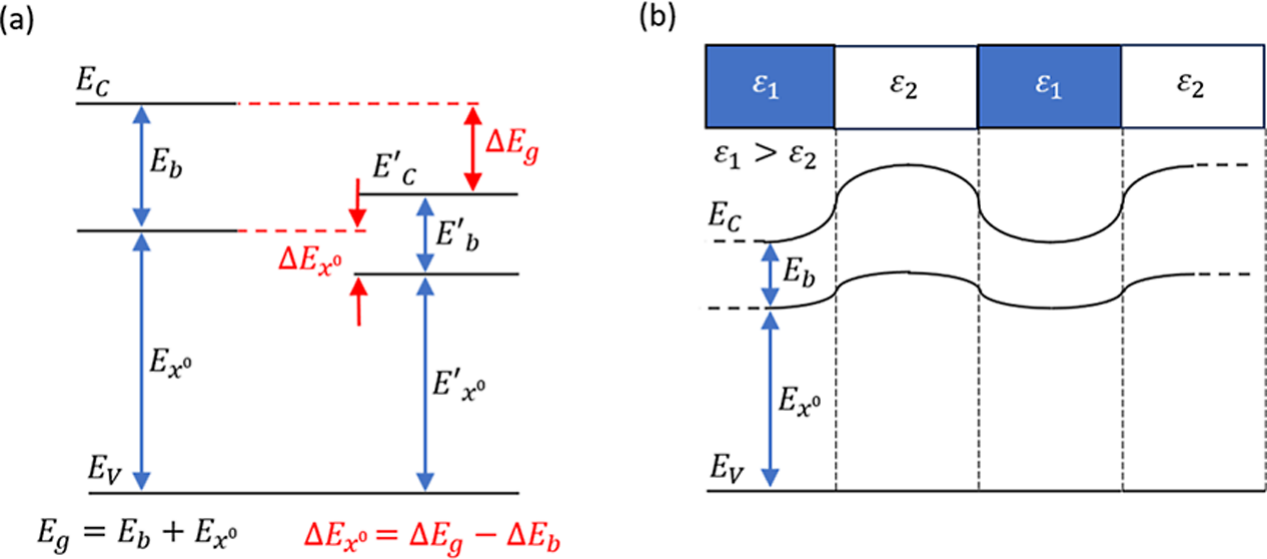
(a) Schematic energy band diagram of the neutral exciton state *X* ^0^ in optically active 2D TMD and undergoing energy modification by the increased dielectric constant of the environment. (b) Schematic illustration of spatial modulation of excitonic properties by variations in the dielectric environment.

Another mechanism for PL manipulation in 2D TMDs involves materials’ doping and changes in charge carriers’ concentration. The characteristic 1L-TMDs PL spectra contain contributions from different excitonic species, including the higher-energy peak associated with the neutral exciton emission (*X* ^0^) and a lower-energy (red-shifted) peak designated to charged excitons (*X* ^+^/*X* ^–^, trions) with a larger binding energy.^25,27,48,49^ Trions are naturally formed in the presence of excess charges and increased doping, which can be stimulated by electrical gating,^25,27,50^ chemical grafting,^51^ laser fluence,^52,53^ or trapped charge defects in the substrate.^40^ Operating at higher carrier densities modifies the PL content by redistributing the neutral-to-charge exciton ratio and enhancing *X* ^+^, *X* ^–^ emission compared to *X* ^0^.^50,53,54^ In this case, one can also expect a strong dependence of the emitted PL content on the type of 2D semiconductors. Specifically, for n-type TMDs (e.g., MoS_2_ and WS_2_), further n-doping would increase electron concentration, forming noticeable *X* ^–^ trion emission, as shown in Figure 2, while for p-type materials (e.g., WSe_2_), increasing n-doping would decrease the hole concentration, resulting in more pronounced *X* ^0^ emission compared to *X* ^+^ trions. Such doping-induced modifications are expected to shift the PL peak position, as shown in Figure 2.

**Figure 2:**
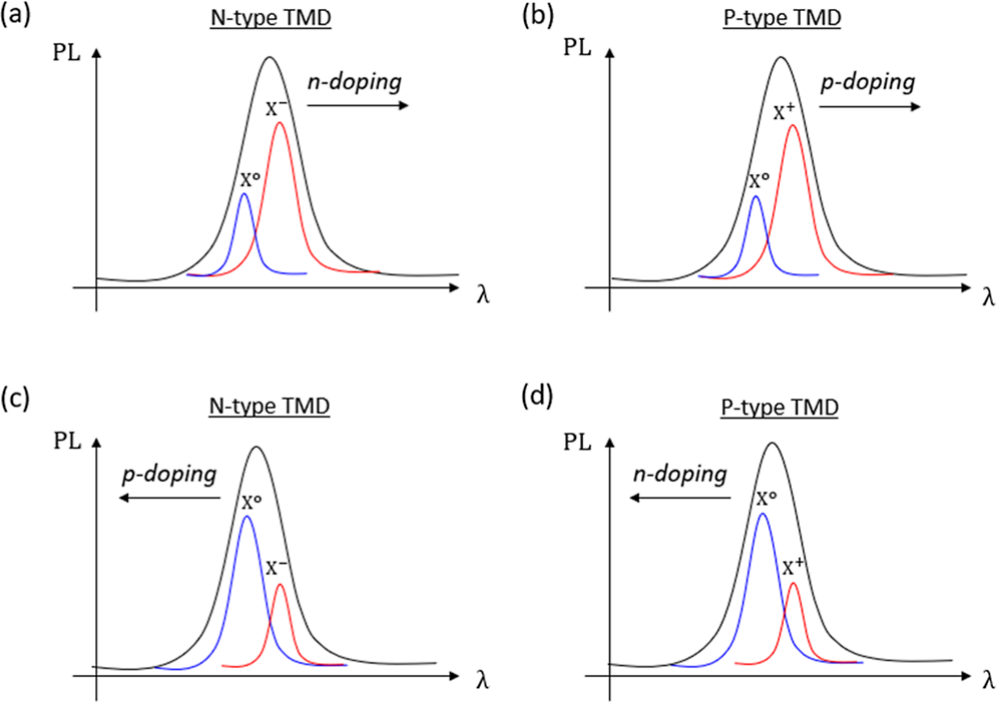
PL spectral shift by doping manipulation and associated modification of the excitonic content in n-type (a), (c) and p-type (b), (d) 2D optically active semiconductors. The blue and the red curves depict the neutral exciton *X* ^0^ and the trions *X* ^+^/*X* ^–^ peaks respectively.

Beyond the PL content modification, higher doping can significantly impact the energy band structure of 2D TMDs.^55,56^ Specifically, increasing carriers’ concentration builds up the screening within the material and leads to the overall bandgap reduction Δ*E*_g_, known as bandgap renormalization (BGR) phenomena.^16,57–59^ The BGR often competes with the changes in binding energy Δ*E*_b_, imprinting complex optical behavior on the excitonic transitions. For the increased doping level (screening) and reduced Coulomb interaction, both the band gap and the binding energies decrease, giving rise to the typically observed redshift in the excitons.^44,60^ However, in some cases, e.g., n-doped monolayers MoS_2_ and WS_2_,^61,62^ it can also show the cancellation effect, i.e., |Δ*E*_g_| ∼ |Δ*E*_b_|, resulting in minimal shifts in excitonic transition energy (Δ*E*_*x*_ = Δ*E*_g_ – Δ*E*_b_ → 0) or even a slight (∼10 meV) blueshift at higher doping concentrations (>10^13^ cm^–2^),^62,63^ alongside the substantial modification of the electronic band structure.

The net effect on optical transitions in 2D TMDs relies on a delicate balance between different phenomena, including semiconductor type and doping, dielectric environment and screening, trapped charges and local gating, carrier densities and band gap renormalization, exciton dynamics, and PL content. Unraveling these complex interactions and controlling the interconnected effects when optically active 2D TMDs are integrated with the CMOS technological platform are crucial for advancing CMOS-compatible TMD-based devices for optoelectronics, sensing, and quantum information processing applications. Although previous works have explored the impact of various substrate and doping effects on 2D TMD PL spectra,^34,35,38–40,43,45,46,51,64^ a dedicated comparison of optical properties between chemical vapor deposition (CVD) grown n- and p-type 2D semiconductors transferred on prepatterned technologically relevant CMOS dielectrics is still missing.

Here, we systematically investigate the interplay between dielectric screening, trap-induced gating, doping, BGR, and excitonic content effects in CVD-grown n-type 1L-WS_2_ and p-type 1L-WSe_2_ transferred on prepatterned chips with different dielectric environments of CMOS-relevant oxides layers (SiO_2_ ε_r_ ∼ 3.9, HfO_2_ ε_r_ ∼ 25). We show that PL spectra are modified differently by combining different 2D semiconductor types coupled to substrate-specific dielectric screening, the doping level, and trapped charges. Specifically, for p-type 1L-WSe_2_, we observe higher PL intensity and a redshift on HfO_2_ compared to the SiO_2_ substrate. At the same time, for n-type 1L-WS_2_, the recorded PL is blue-shifted and shows a lower intensity for a similar dielectric environment. The difference arises from the interplay between semiconductor type, doping, dielectric screening, and charge carrier concentration. We also demonstrate that removing the substrate and suspending TMD monolayers at the nanoscale dramatically reduces the nonradiative recombination pathway, resulting in spatially confined enhanced PL. We show that higher-intensity emission hot spots can be simultaneously controlled and deterministically localized on the chip by substrate engineering of a dielectric environment. Our findings in this work contribute to unraveling the essential excitonic effects in optically active 2D TMDs integrated with CMOS-relevant dielectrics, taking a step forward toward realizing the full potential of 2D semiconductors in next-generation 2D CMOS-compatible optoelectronic devices.

## Results and Discussion

To investigate the influence of CMOS-dielectrics on optoelectronic properties of 2D TMDs, a 35 nm thick high-k HfO_2_ layer was deposited onto 90 nm thick SiO_2_ by using atomic layer deposition (ALD). The samples were patterned using e-beam lithography followed by reactive ion etching (RIE) of HfO_2_ to expose SiO_2_ areas and realize different dielectric environments on the same chip for 2D materials transfer (see Methods). CVD-grown 1L-WS_2_ and 1L-WSe_2_ were then transferred onto prepatterned substrates using a semidry vacuum-assisted transfer method (see Methods). Figures 3a and 4a show the optical microscopy images of the transferred 1L-WSe_2_ and 1L-WS_2_ triangular flakes covering both SiO_2_ and HfO_2_ areas. The exposed SiO_2_ region, tens of micrometers in size, and the shallow (∼40 nm) etch depth of the top HfO_2_ layer minimize strain effects on the flake during the transfer and consequent PL measurements. To monitor the quality of the TMD transfer process, we performed Raman spectroscopy characterizations of the as-grown materials on SiO_2_ and after the transfer to the target chip (see Supporting Information). The Raman spectra of the transferred 1L-WS_2_ and 1L-WSe_2_ measured on the SiO_2_ regions show negligible variations compared to the as-grown materials, indicating that no significant material degradation, additional defects, doping changes (polymer residues), or strain were introduced during the transfer process.

**Figure 3:**
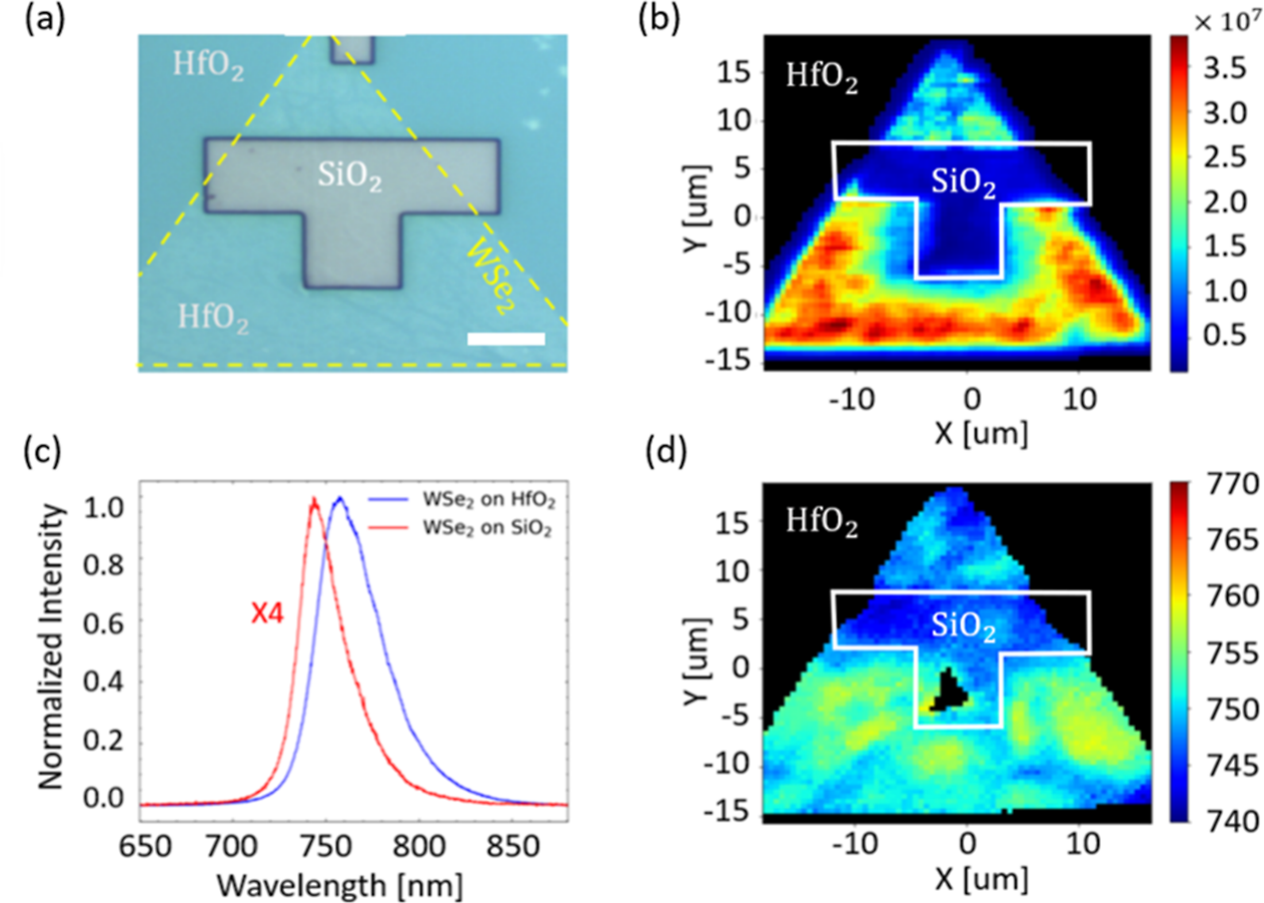
(a) Optical microscopy image of 1L-WSe_2_ flake (yellow dashed contour) transferred on a prepatterned chip with SiO_2_ and HfO_2_ areas. The scale bar is 10 μm. (b) Integral PL intensity map of 1L-WSe_2_ flake covering both SiO_2_ and HfO_2_ substrates. (c) Representative PL spectra of 1L-WSe_2_ on SiO_2_ and HfO_2_ areas. (d) PL spectral map of 1L-WSe_2_ on SiO_2_ and HfO_2_ areas.

**Figure 4:**
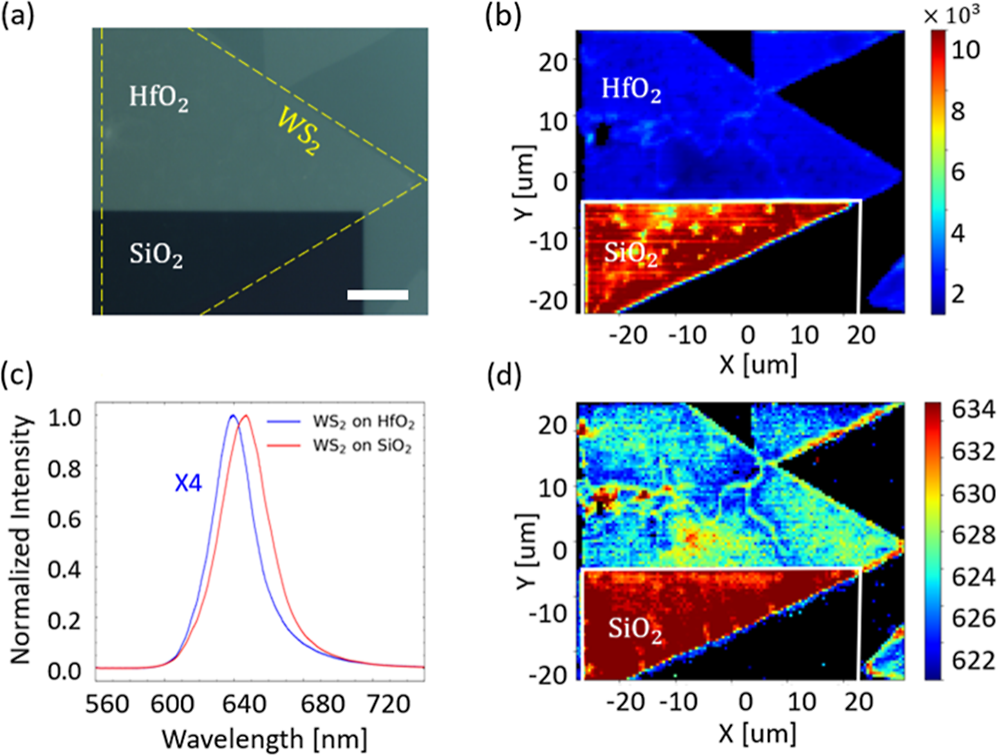
(a) Optical microscopy image of the 1L-WS_2_ flake (yellow dashed contour) transferred onto a prepatterned chip with exposed SiO_2_ and HfO_2_ areas. The scale bar is 10 μm. (b) Integral PL intensity map of the 1L-WS_2_ flake covering both the SiO_2_ and HfO_2_ substrates. (c) Representative PL spectra of the 1L-WS_2_ on SiO_2_ and HfO_2_ areas. (d) PL spectral map of 1L-WS_2_ on SiO_2_ and HfO_2_ areas.

The PL spectra, the integrated PL intensity, and peak position maps were acquired at room temperature in ambient conditions (see Methods), using excitation laser intensity below ∼5 kW/cm^2^ to minimize heating and parasitic thermal effects.^65^ We first confirmed the reproducibility and consistency of the TMDs optical response (i.e., PL shape, peak position, intensity, and spectral line width) across multiple (∼10) CVD-grown flakes transferred altogether in the same process to the same prepatterned chip (see Supporting Information). All the measurements on different samples were conducted using identical experimental conditions, and the PL spectra were collected using the same data acquisition procedure (see Methods). The measured PL spectra across different samples exhibited consistent spectral features (Supporting Information), indicating the reproducibility and robustness of the PL data, high uniformity of 2D monolayers under test, and excellent quality of the transfer process. The integrated PL intensity and peak position maps of 1L-WSe_2_ are shown in Figure 3b,d, respectively, revealing PL variations across the flake in different dielectric regions. We observed a narrower PL line width with a notably four-times lower intensity in the area where 1L-WSe_2_ is in contact with the SiO_2_ substrate (Figure 3b,c), and a red-shifted (∼30 meV, ∼14 nm), broader PL peak with higher intensity on the high-k HfO_2_ region (Figure 3b,d).

The observed PL redshift and broadening (Figure 3c) are ascribed to increased dielectric screening, which reduces the exciton binding energy and the semiconductor bandgap. This effect is stimulated by the higher dielectric permittivity (ε_r_ ∼ 25) of the HfO_2_ substrate compared to SiO_2_.^46^ The broader PL line width is also related to the higher p-doping of 1L-WSe_2_ on HfO_2_ induced by the structural defects and electron trap charges due to oxygen deficiency in hafnia.^66^ The latter negatively gates (i.e., holes injection) the 2D layer on top and brings a more pronounced, spectrally shifted contribution of *X* ^+^ trions that lead to broader PL spectra.^50^ On the other hand, the narrower PL peak on SiO_2_ is related to lower p-doping due to electrons’ injection from a positive gating by trap charges at the Si/SiO_2_ interface.^67^ The 1L-WSe_2_ PL spectral inhomogeneity on HfO_2_ (Figure 3d) is attributed to local electrostatic gating and possible strain variations within the flake, resulting from the larger surface roughness of the ALD-made HfO_2_ compared to the thermally grown SiO_2_.^68,69^

The same analyses were carried out on the 1L-WS_2_ flake transferred to another SiO_2_/HfO_2_ area, as shown in Figure 4a. The recorded PL spectra on HfO_2_ are blue-shifted (∼22.5 meV, ∼7.5 nm), showing four times lower intensity (Figure 4b,c) than on SiO_2_. The observed blue shift is attributed to BGR in 1L-WS_2,_ where the energy bandgap and exciton binding energy are reduced on a higher permittivity HfO_2_ substrate, with the exciton binding energy experiencing a greater reduction.^70^ Additional contribution to the blue shift arises from a reduced *X* ^–^ trion emission and its smaller contribution to the PL content since negatively charged traps in HfO_2_ act as local gates and decrease the 1L-WS_2_ n-doping (Figure 2c).

To gain deeper insights and validate the origin of the observed PL changes, we decomposed the recorded PL spectra on SiO_2_ and HfO_2_ substrates into two excitonic contributions (*X* ^0^ and *X* ^+^, *X* ^–^) using a double-Lorentzian fit. The fitting procedure and error residue analysis are presented in the Supporting Information. For the 1L-WS_2_/SiO_2_ interface, the fitted *X* ^–^ trion peak is located at ∼647.2 nm (∼1.915 eV, red curve), and it overcomes the *X* ^0^ exciton centered at ∼635.8 nm (∼1.95 eV, blue curve), as shown in Figure 5a. The spectral weight, i.e., the integral intensity (area under the curve) of the emission peak, *A*_*X*_, is proportional to the relevant exciton population *N*_*X*_.^48,71^ Specifically, the ratio between the trions *A*_*X*_^–^ and neutral excitons *A*_*X*_^0^ spectral weights and their relative contributions to the overall PL signal relates the information about the free carriers concentration in optically active 2D semiconductors.^51,72^ Namely, *A*_*X*_^–^/*A*_*X*_^0^ ∝ *N*_*X*_^–^/*N*_*X*_^0^ and *N*_*X*_^–^ = Κ_T_·*N*_*X*_^0^·*n*_e_, where *N*_*X*_^–^ and *N*_*X*_^0^ are the trions *X* ^–^ and excitons *X* ^0^ concentrations, Κ_T_ is the rate for exciton-to-trion conversion, and *n*_e_ is the free charge carriers (e.g., electrons) concentration. For 1L-WS_2_ on SiO_2_, we get the normalized *A*_*X*_^–^ ∼ 0.63 and *A*_*X*_^0^ ∼ 0.37 and exciton ratio of *A*_*X*_^–^/*A*_*X*_^0^ ∼ 1.7, showing the trions’ spectral weight prevails over the neutral excitons. The latter is consistent with the expected n-doping increase on SiO_2_ induced by the electrostatic gating of positive trap charges. Furthermore, the PL fit of 1L-WS_2_ on HfO_2_ (Figure 5a) reveals modified excitonic content showing a slightly blue-shifted *X* ^–^ trion located at ∼645.3 nm (∼1.921 eV, red curve) with reduced spectral weight *A*_*X*_^–^ ∼ 0.57, and *X* ^0^ exciton peak centered at ∼635.5 nm (∼1.951 eV, blue curve) with increased contribution *A*_*X*_^0^ ∼ 0.43 (*A*_*X*_^–^/*A*_*X*_^0^ ∼ 1.33) to PL content compared to the SiO_2_ substrate. A minor blue shift is attributed to BGR in n-doped 1L-WS_2_,^70,73^ while the reduced *X* ^–^ (increased *X* ^0^) spectral weights originate from the lower n-doping in 1L-WS_2_ induced by HfO_2_ traps. Another noticeable effect evident from PL deconvolution is related to the trion binding energy. Specifically, the calculated energy separations between *X* ^–^ and *X* ^0^ peaks on SiO_2_ and HfO_2_ are ∼35 meV and ∼43 meV, respectively. This increase is related to the screening effect of the dielectric environment, where larger-sized charged excitons experience stronger dielectric screening on a higher permittivity substrate compared to neutral excitons. As a result, the *X* ^–^ binding energy on HfO_2_ is reduced more compared to that on *X* ^0^, showing a larger energy separation.

**Figure 5:**
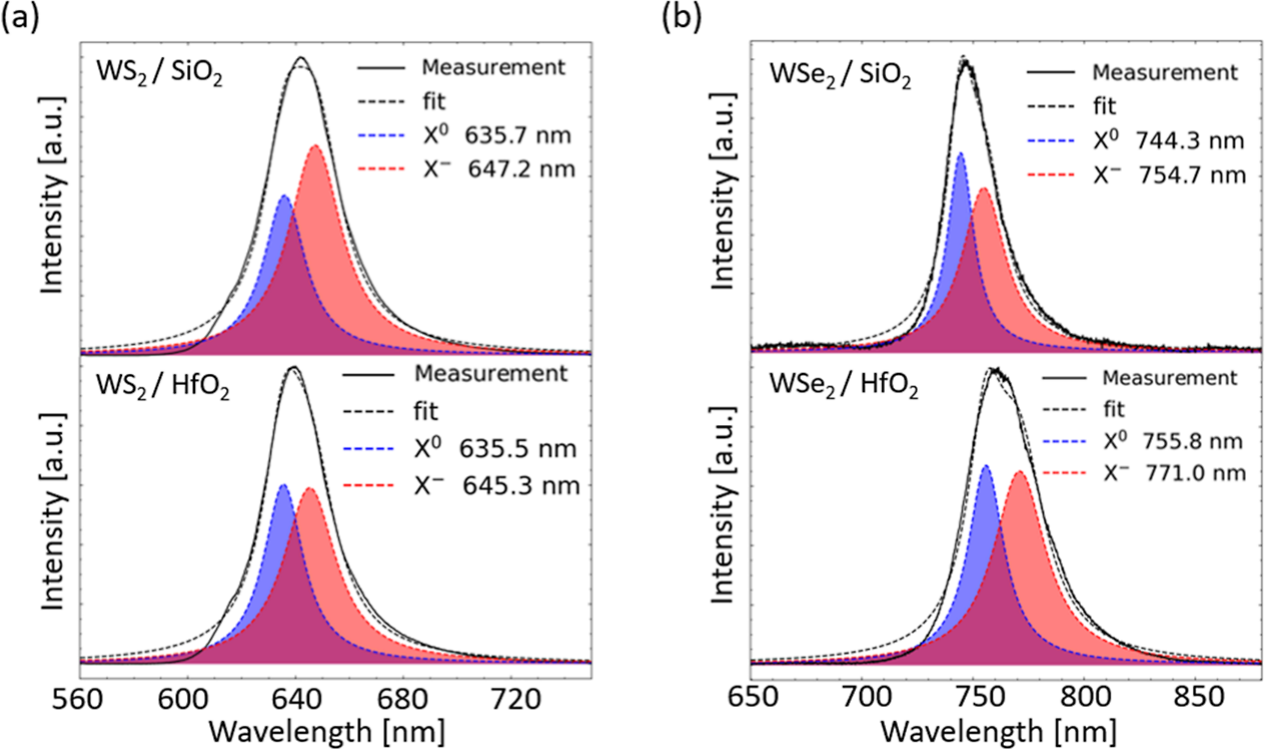
PL spectra decomposition using double Lorentzian fit. (a) 1L-WS_2_ on SiO_2_ and HfO_2_ substrates and (b) 1L-WSe_2_ on SiO_2_ and HfO_2_. The blue and the red curves indicate the fitted neutral exciton *X* ^0^ and trions *X* ^+^/*X* ^–^ peaks, respectively.

Similarly, we fitted the PL spectra of the transferred 1L-WSe_2_, as shown in Figure 5b. For the 1L-WSe_2_/SiO_2_ interface, we get the *X* ^+^ trion peak positioned at ∼754.7 nm (∼1.643 eV, red curve) and the *X* ^0^ exciton peak at ∼744.3 nm (∼1.665 eV, blue curve). Like in the 1L-WS_2_ case, here the spectral weight of trions *A*_*X*_^+^ ∼ 0.57 exceeds one of the excitons *A*_*X*_^0^ ∼ 0.43 (*A*_*X*_^+^/*A*_*X*_^0^ ∼ 1.33), which can imply the presence of material defects in our CVD 2D layers. The narrower PL line width and higher *X* ^0^ peak intensity compared to *X* ^+^ trions (Figure 5b) indicates the reduced p-doping of 1L-WSe_2_ on SiO_2_ due to the positive gating from the oxide trap charges. On the other hand, the 1L-WSe_2_ PL measurements on HfO_2_ show a pronounced red-shift due to the dielectric screening effect, with *X* ^+^ and *X* ^0^ peaks shifted to longer wavelengths at ∼771.1 nm (∼1.608 eV, red curve) and ∼755.8 nm (∼1.641 eV, blue curve), respectively. In addition to screening, the negative gating by HfO_2_ traps increases the 1L-WSe_2_ p-doping and the corresponding *X* ^+^ trion peak intensity, contributing to broader PL line width and modified spectral weights *A*_*X*_^+^ ∼ 0.58 and *A*_*X*_^0^ ∼ 0.42 (*A*_*X*_^+^/*A*_*X*_^0^ ∼ 1.38). Similar to 1L-WS_2_, we got the increased energy separation between *X* ^+^ and *X* ^0^ peaks in1L-WSe_2_, i.e., from ∼23 meV on SiO_2_ to ∼30 meV on HfO_2_, due to stronger trion interactions and screening induced by the dielectric environment.

It should be noted in this context that the different dielectric environments (SiO_2_ and HfO_2_ substrates) used in our experiments accommodate different trap charges that locally gate the 2D layers on top, changing their doping concentration. The latter modifies the intensities of the *X* ^0^ and *X* ^+^, *X* ^–^ peaks, as presented in Figure 5. However, based on PL fitting, we recognize that the associated spectral weights (*A*_*X*_^0^, *A*_*X*_^–^, and *A*_*X*_^+^) in both 1L-WS_2_ and 1L-WSe_2_ demonstrate only limited (<10%) modulation depth in response to the doping variations induced by the substrate. Therefore, the observed PL intensity quenching (Figures 3c and 4c) is not fully correlated to the materials’ free carrier concentration and/or excitonic content (*X* ^0^ vs *X* ^+^, *X* ^–^) and likely results from the enhanced nonradiative recombination pathways at 1L-WSe_2_/SiO_2_ and 1L-WS_2_/HfO_2_ interfaces invoked by interfacial defects, trapped impurities, surface-activated exciton scatters, or local strain.

To exploit the observed PL intensity contrast and demonstrate PL patterning by the engineered dielectric environment, we transferred 1L-WSe_2_ on prepatterned hole arrays of different diameters (Figure 6) and mapped the PL spectra across the flake. Figure 6b,e,h shows the integrated PL intensity maps collected from the sample. As expected, we observed the reduced PL over the larger (5 and 3 μm) holes where 1L-WSe_2_ is supposedly in contact with the SiO_2_ substrate (Figure 6b,e). One can notice that both the PL intensity and peak position nicely reproduce the geometric (holes) pattern of the substrate. We also recognized some areas of enhanced PL having a form of “red semi-circles” that can be attributed to the edge localization and scattering effects from the holes’ boundaries (Figure 6b,e). Additionally, the reduced PL intensity at the center of the flakes can be assigned to nucleation sites and multilayer islands formed during the CVD growth.^74^

**Figure 6:**
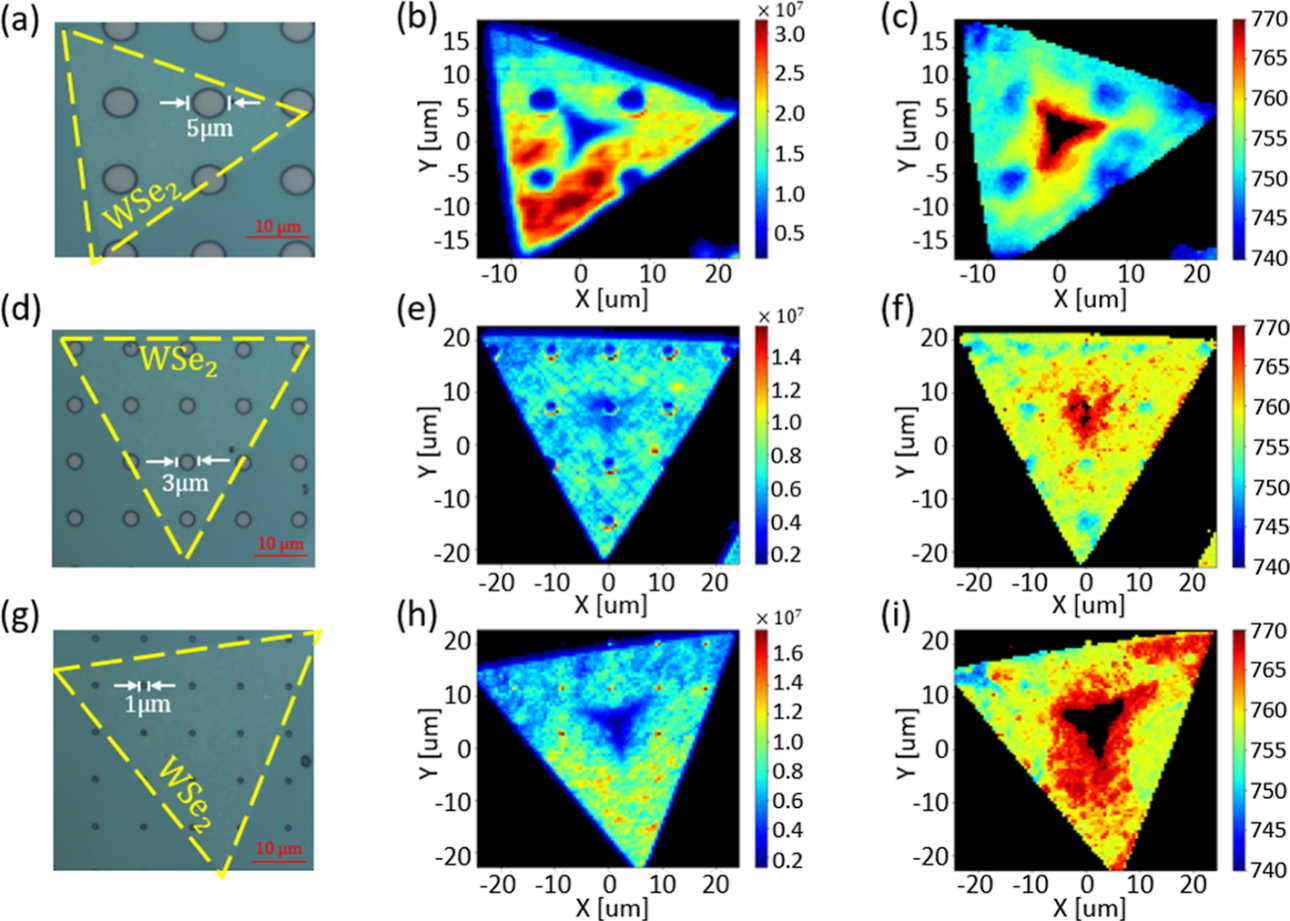
PL study of 1L-WSe_2_ on prepatterned SiO_2_ holes of different diameters surrounded by a high permittivity HfO_2_ layer. (a,d,g) Optical microscopy images of the fabricated devices with 1L-WSe_2_ transferred on SiO_2_ holes with 5 μm, 3 μm, and 1 μm diameters, respectively. (b,e,h) PL intensity map of 1L-WSe_2_ transferred on SiO_2_ holes with 5 μm, 3 μm, and 1 μm diameters, respectively. (c,f,i) PL spectral map of 1L-WSe_2_ transferred on SiO_2_ holes with 5 μm, 3 μm, and 1 μm diameters, respectively.

In the case of the smaller hole array (i.e., 1 μm), we measured higher PL intensities above the holes (Figure 6h) compared to the background collected from the HfO_2_ areas. This can indicate that the 1L-WSe_2_ is no longer in contact with SiO_2_, and the flake is suspended above the holes, showing stronger PL due to suppressed nonradiative recombination pathways related to the substrate.^40^ Another possible contribution to higher PL intensity may arise from the edge effect, where the diffraction-limited optical system can artificially enhance signals near the hole boundaries, leading to localized intensity peaks. To get further insight into the origin of PL enhancement, we performed scanning electron microscopy (SEM) characterizations of TMD flakes transferred on different hole arrays (see Supporting Information). We verified that for larger hole diameters (5 and 3 μm), the 1L-WSe_2_ is laid down into the hole and is in contact with the SiO_2_ substrate. In comparison, for smaller diameters (<1 μm), the monolayer seems to be suspended above the hole, blurring the SEM signal from the bottom (SiO_2_) of the hole area. Implying the suspended nature of the monolayer from the SEM characterization (see Supporting Information), we expect the membrane-like behavior of 1L-WSe_2_ with the suppressed nonradiative recombination pathway to play a significant role in the observed localized PL enhancement (Figure 6h), compared with edge scattering effects.

The PL decomposition from the signals recorded over different hole areas (Figure 7) reveals trends similar to those previously discussed regarding the spectral properties, excitonic content, and spectral weight of the emitted radiation. Specifically, the collected PL from a 5 μm hole, where the flake is in contact with SiO_2_, shows a higher intensity *X* ^0^ peak at ∼748.2 nm (∼1.657 eV, blue curve) compared to that of the *X* ^+^ located at ∼762.4 nm (∼1.626 eV, red curve). The calculated spectral weights *A*_*X*_^+^ ∼ 0.49 and *A*_*X*_^0^ ∼ 0.51 (*A*_*X*_^+^/*A*_*X*_^0^ ∼ 0.96) indicate lower p-doping of 1L-WSe_2_ on SiO_2_. When the hole diameter decreases to 3 μm, the PL intensity from the holes’ areas shows only a minor variation (Figure 7b), demonstrating that the flake is still in contact with the SiO_2_ substrate, resulting in reduced PL intensity from the holes’ area. Nevertheless, broader PL line width (for 3 μm holes) and modified spectral content *A*_*X*_^+^ ∼ 0.55 and *A*_*X*_^0^ ∼ 0.45 (*A*_*X*_^+^/*A*_*X*_^0^ ∼ 1.22) with increased trion contribution compared to a 5 μm hole diameter suggests higher 1L-WSe_2_ doping and stronger influence of the surrounding HfO_2_ area. The PL spectra collected from the 1 μm holes show a distinct red-shift and spectral broadening (Figure 7c), signifying the major influence of the surrounding HfO_2_ substrate on the measured PL properties. Namely, the observed PL red-shift with the *X* ^0^ peak position at ∼756.4 nm (∼1.64 eV, blue curve) and *X* ^+^ peak centered at ∼774.3 nm (∼1.61 eV, red curve) demonstrates a pronounced effect of dielectric screening and/or doping level imposed by HfO_2_. The latter is also reinforced by further increase of the trion peak *A*_*X*_^+^ ∼ 0.58 and spectral weight ratio *A*_*X*_^+^/*A*_*X*_^0^ ∼ 1.27, demonstrating higher p-doping of 1L-WSe_2_ when mainly supported by the HfO_2_ substrate.

**Figure 7:**
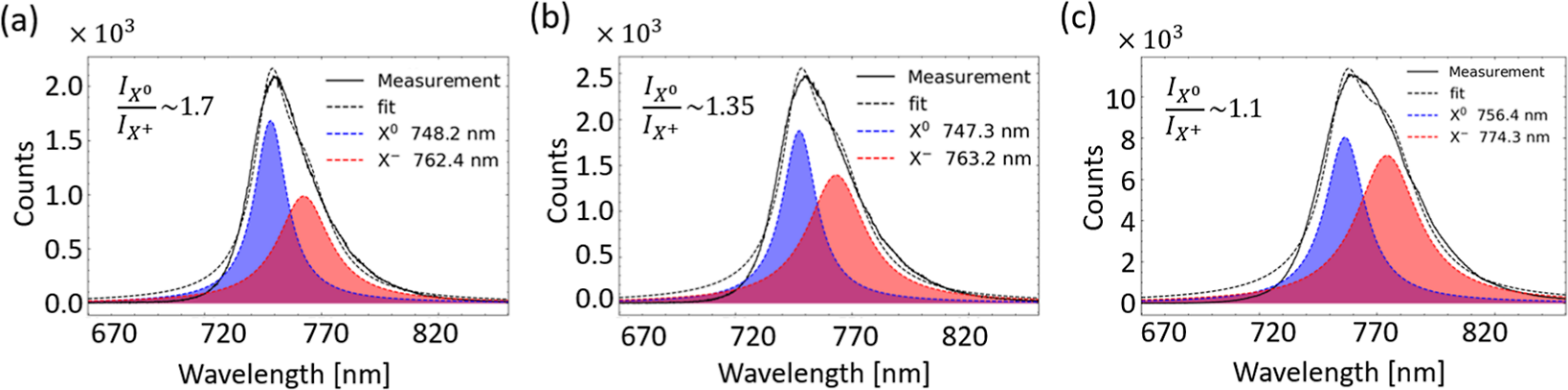
PL spectra decomposition corresponding to each hole size of (a) 5 μm, (b) 3 μm, and (c) 1 μm using double Lorentzian fit. The blue and the red curves indicate the fitted neutral exciton *X* ^0^ and *X* ^+^ trion peaks, respectively. The *I*_*X*_^0^/*I*_*X*_^+^ indicate the exciton-to-trion intensity ratio. For smaller hole diameters, the measured PL is significantly controlled by the properties of the surrounding HfO_2_ substrate.

Finally, taking advantage of the observed enhancement of PL intensity from smaller hole diameters and suspended 2D TMD emitters, we engineered a spatially localized and enhanced emission from a micro-PL array of closely packed 500 and 250 nm diameter holes with transferred 1L-WSe_2_ on top, as shown in Figure 8. The corresponding PL intensity maps plotted at 750 nm wavelength (Figure 8b,d) highlight nanoscale PL hot spots from the deterministic hole areas, demonstrating PL enhancement factors of 2.25 and 1.5 for the 500 and 250 nm hole diameters, respectively.

**Figure 8:**
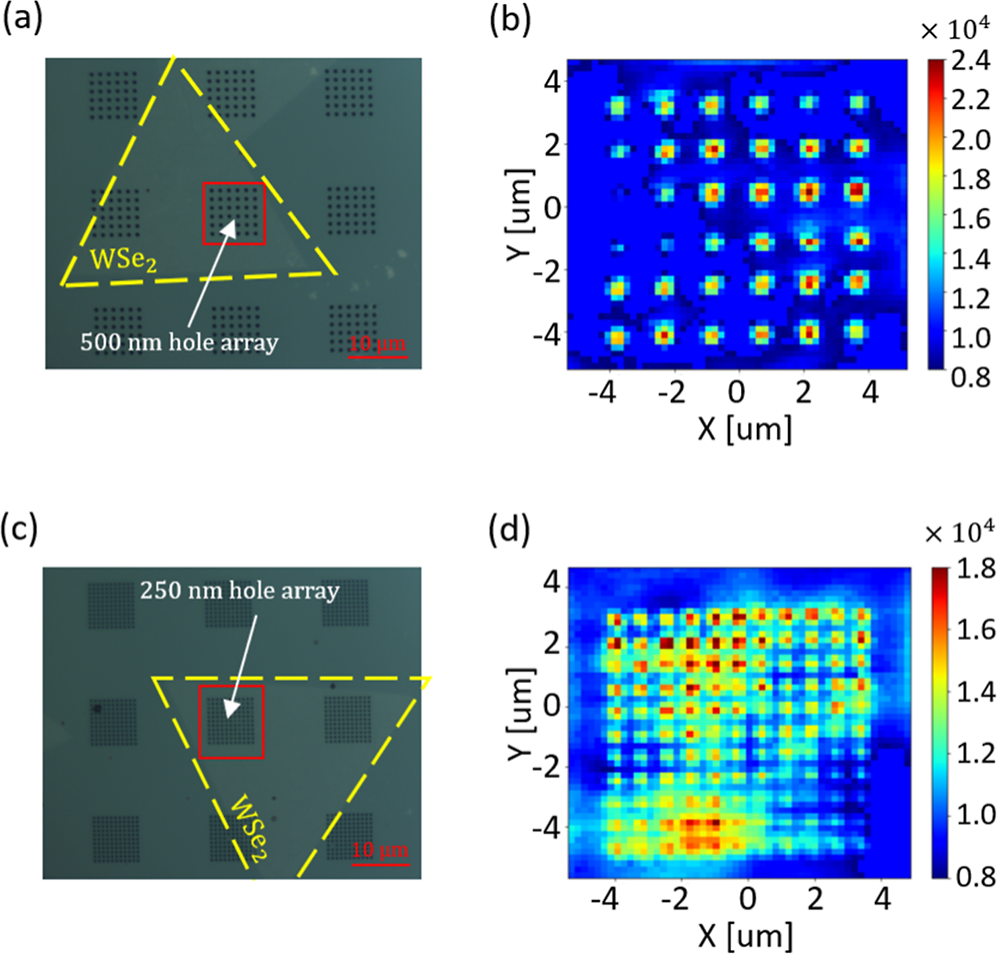
Micro-PL array using 1L-WSe_2_ flake transferred on prepatterned densely packed holes of 500 and 250 nm diameters. (a) Optical microscope image of 1L-WSe_2_ transferred on a 500 nm diameter hole array. (b) PL intensity map at 750 nm wavelength was collected over the area of a 500 nm hole array. (c) Optical microscopy image of 1L-WSe_2_ transferred on a 250 nm diameter hole array. (d) PL intensity map at 750 nm wavelength was collected over the area of a 250 nm hole array.

## Conclusion

In summary, we investigated the PL properties of CVD 1L-WS_2_ and 1L-WSe_2_ 2D semiconductors semidry transferred on CMOS-relevant SiO_2_ and HfO_2_ dielectric materials with dissimilar dielectric permittivity and trap charges. The measured PL spectra are modified differently for n- and p-type 2D layers, interacting with substrate dielectric screening, defects, and the material doping level. For p-type 1L-WSe_2_, we observed higher PL intensity and red-shifted trion-dominated emission on a high dielectric HfO_2_ substrate with an increased p-doping compared to the SiO_2_. For n-type 1L-WS_2_, the recorded PL is blue-shifted at a lower intensity in a similar dielectric environment. We exploited the PL intensity contrast and demonstrated PL patterning by an engineered dielectric environment. We demonstrated that removing the substrate and suspending 1L-WSe_2_ on a nanometer (250 nm, 500 nm) scale dramatically reduces the nonradiative recombination pathway, resulting in spatially confined enhanced PL. We demonstrated micro-PL arrays and verified that higher-intensity localized emission hot spots can be spatially controlled and engineered using a dielectric environment. Our findings contribute to unraveling the essential excitonic effects in 2D TMDs toward their potential technological application in advanced CMOS-compatible optoelectronic devices.

## Methods

As a substrate, we used a commercial Si wafer with a 90 nm thick thermally grown SiO_2_ layer and a 35 nm thick HfO_2_ layer deposited on top by ALD. The modification of the dielectric environment and holes’ patterning were realized by electron beam lithography using a RAITH EBPG 5200 writer and AR-P 6200 series (CSAR) e-beam resist. The pattern was transferred to HfO_2_ and SiO_2_ layers by using RIE etch with a CF_4_/O_2_ gas mixture.

As for the transfer, the chips with CVD-grown 1L-WS_2_ and 1L-WSe_2_ were coated with polystyrene (PS) and immersed in deionized (DI) water. The PS/1L-TMD stack was picked up, washed, dried, and laminated onto a prepatterned chip pretreated with the O_2_ plasma. After the transfer, the PS support layer was stripped with toluene, exposing 1L-WS_2_ and 1L-WSe_2_ for further processing.

PL spectra were acquired using a Horiba LabRAM HR Evolution spectrometer at an excitation wavelength of 532 nm and a laser power of <100 μW (<5 kW/cm^2^) to avoid sample heating or oxidation in air. An integration time of 0.5 s and 2 accumulations were used for PL measurements. The laser beam was focused onto the sample by using a 100× objective lens with an NA of 0.9. The scattered light was collected and collimated with the same lens. The scattered signal was dispersed by a diffraction grating with 900 grooves/mm and then detected by a thermoelectrically cooled CCD (charge-coupled device) detector at −60 °C. All of the PL spectra were recorded for the same integration time, laser power, and focus conditions.

## Supplementary Material



## Notes

Spectroscopic analysis of Raman and PL spectra, SEM characterization, PL fitting, and error analysis (PDF)
