# Supplementary material for: Exciton Manipulation via Dielectric Environment Engineering in 2D Semiconductors
Source: ACS Appl Opt Mater. 2025 May 20;3(6):1330–8. doi: 10.1021/acsaom.5c00105 (PMC12210256; doi:10.1021/acsaom.5c00105)
Supplement: Supplementary file 1 [file ot5c00105_si_001.pdf]

Supporting Information

Exciton manipulation via dielectric environment engineering in 2D semiconductors

Raziel Itzhak[1,2], Nathan Suleymanov[1], Boris Minkovich[1], Liana Kartvelishvili[2], Vladislav Kostianovski[2], Roman Korobko[2], Alex Hayat[1], and Ilya Goykhman[2]*

[1] Department of Electrical and Computer Engineering, Technion, Haifa, Israel.

[2] Institute of Applied Physics, The Faculty of Science and The Center for Nanoscience and Nanotechnology, The Hebrew University of Jerusalem, Jerusalem 91904, Israel.

KEYWORDS 2D materials, monolayer TMDs, excitons, dielectric environment, binding energy, photoluminescence, nano-optoelectronics.

Corresponding Author

Ilya Goykhman, email: ilya.goykhman@mail.huji.ac.il

Raman Characterization

To assess the quality of the TMD layers, we performed Raman spectroscopy characterizations of the as-grown material on SiO_2 and after the transfer. Figure S1a shows the Raman spectra of CVD-grown WS_2 on SiO_2 under 532 nm excitation, before (red curve) and after (blue curve) the transfer. The peak at ~355 cm^{-1} corresponds to the in-plane (E^1_{2g}) vibrational mode, while the peak at ~417.2 cm^{-1} corresponds to the out-of-plane (A_{1g}) mode [1–3]. The E^1_{2g} mode softens and the A_{1g} mode stiffens with increasing layer thickness [1,2], thus, the frequency difference between these modes can be used to determine the number of layers [1,2]. The measured peak separation of ~65 cm^{-1} confirms that monolayer WS_2 (1L-WS_2) was used in our process[1]. Similarly, Figure S1b presents the Raman spectra of CVD-grown WSe_2 on SiO_2 under 532 nm excitation. The broad peak at ~260 cm^{-1} corresponds to the overlayed peaks of E^1_{2g} and A_{1g} modes of monolayer WSe_2 at room temperature[1]. As evident, the Raman spectra after the transfer on SiO2 substrate of 1L-WS_2 and 1L-WSe_2 show negligible variations compared to the as-grown materials. This indicates that no significant material degradation, material defects, polymer residues with associated doping changes, or additional strain were introduced during the transfer process.

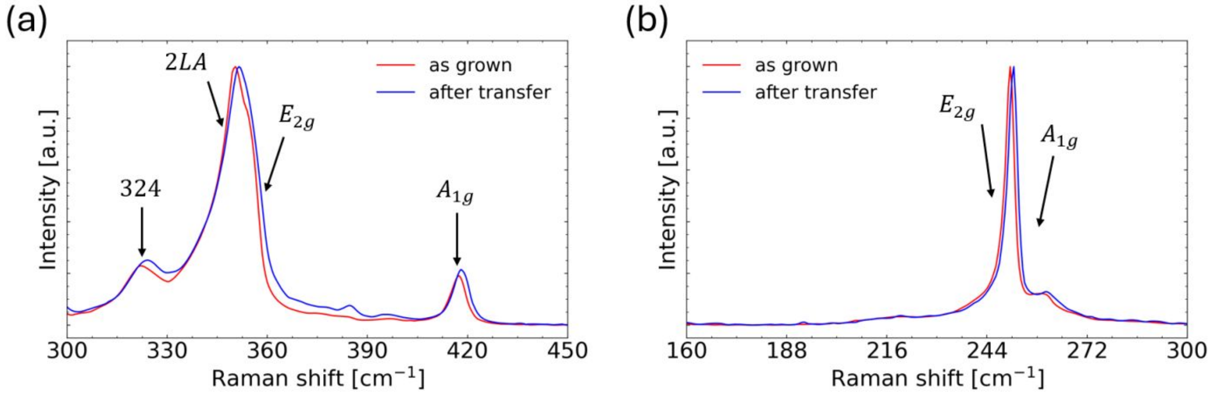

Figure S1. Raman spectra of (a) 1L-WS_2 and (b) 1L-WSe_2 before and after transfer, collected under excitation with a 532 nm laser.

Reproducibility of the PL Spectra

The reproducibility and consistency of the PL spectra were compared across multiple (~10) CVD flakes transferred altogether in the same process to the same prepatterned chip. All the measurements on different samples were conducted using identical experimental conditions, and the PL spectra were collected using the same data acquisition procedure. The representative data of multiple PL spectra acquired on different samples (e.g. 1L-WS_2 flakes) are shown in Figure S2:

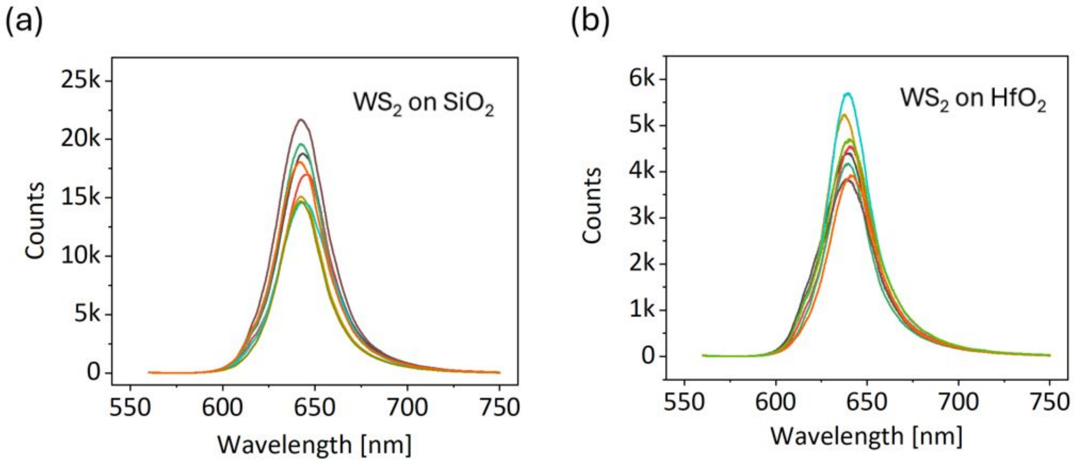

Figure S2. Representative PL spectra distribution acquired across multiple 1L-WS_2 flakes on (a) SiO_2 and (b) HfO_2 substrates.

The measured PL spectra exhibited consistent spectral features across multiple samples (i.e., peak position, PL intensities, line shapes), indicating the reproducibility of the PL data, its robustness and strong evidence of the investigated physical phenomena, as well as high uniformity of 2D monolayers under test, and exceptional quality of our transfer method. The statistical analysis of the peak position, intensity, and full width at half maximum (FWHM) revealed only minor variations (< 10%) among the different samples, further confirming the high degree of uniformity of the acquired PL data across the different flakes. For instance, we found the mean PL peak position of 1L-WS2 samples to be 645.5 ± 1.8 nm on SiO_2 and 639.1 ± 1.4 nm on HfO_2 substrates. The corresponding mean PL intensities were 17,681 ± 1,060 counts and 4,473 ± 421 counts, respectively. The average PL linewidth FWHM was estimated to be 31.4 ± 1.8 nm and 29.2 ± 1.4 nm on SiO_2 and HfO_2 substrates, respectively. These results indicate that the PL peak position on SiO_2 exhibits a blue shift of 6.4 nm (~19.5 meV) compared to that on HfO_2, along with an intensity enhancement of ~3.95, confirming the reproducibility of the results obtained from the PL map on a single flake (main text, page 10) across multiple samples on the same chip.

SEM Characterization

To validate the actual structure of the transferred 1L-WSe2 flakes on hole arrays of different diameters, we performed the SEM characterizations of the fabricated samples. The SEM micrographs (false color) are shown in Figure S3.

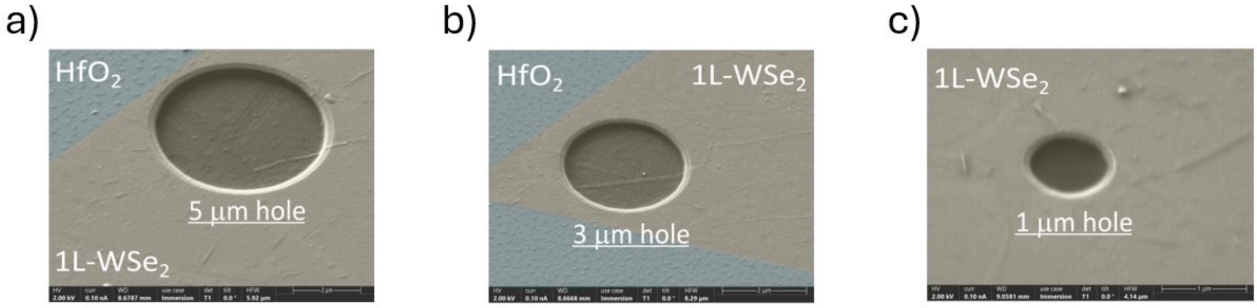

Figure S3. Scanning electron microscope (SEM) micrographs (false color) of the transferred 1L-WSe2 flakes on a) 5 μm diameter holes array, b) 3 μm diameter holes array, and c) 1 μm diameter holes array.

As evident from Figures S3a and S3b, the 1L-WSe2 is laid down inside the 5 μm and 3 μm holes, showing a clear contact with the SiO2 substrate. In contrast, for 1 μm diameter (Figure S3c) the monolayer seems suspended above the hole, blurring the SEM signal from the bottom SiO2 hole area.

PL Fitting and Error Analysis

The fitting procedure was conducted as follows: initial parameters were chosen based on values previously reported in the literature[4–9] for specific substrate, and subsequently the fit was optimized to minimize the fitting error under the following restriction: The peak position of the neutral exciton (X^0) was constrained within a narrow range of ±2 nm, and the charged exciton (X^-) was initially set to be redshifted ~10 nm (~30 meV) relative to the neutral exciton[6]. Other parameters, such as peak intensity and full width at half maximum (FWHM), were allowed to vary and were optimized during the fitting process. This approach consistently reproduced the same spectral trends and peak characteristics across all samples, confirming the robustness of the analysis.

Figures S4a and S4c show the decomposition of the PL spectra using a double Lorentzian fit for 1L-WS_2 on SiO_2 and HfO_2 substrates, respectively. Figures S4b and S4d present the

corresponding residuals between the experimental data and the fitted curves. The fitting error was quantified using the root mean squared error (RMSE), yielding values of 0.025 ± 0.002 for the SiO_2 substrate and 0.027 ± 0.003 for the HfO_2 substrate. The energy separation between the X^0 and X^- peaks was found to be 11.5 ± 0.7 nm for 1L-WS_2 on SiO_2 and 11 ± 1 nm on HfO_2. The area ratio between the peaks, defined as A_{X^-}/A_{X^0}, was approximately 2 ± 0.3 for WS_2 on SiO_2 and 1.6 ± 0.3 for WS_2 on HfO_2.

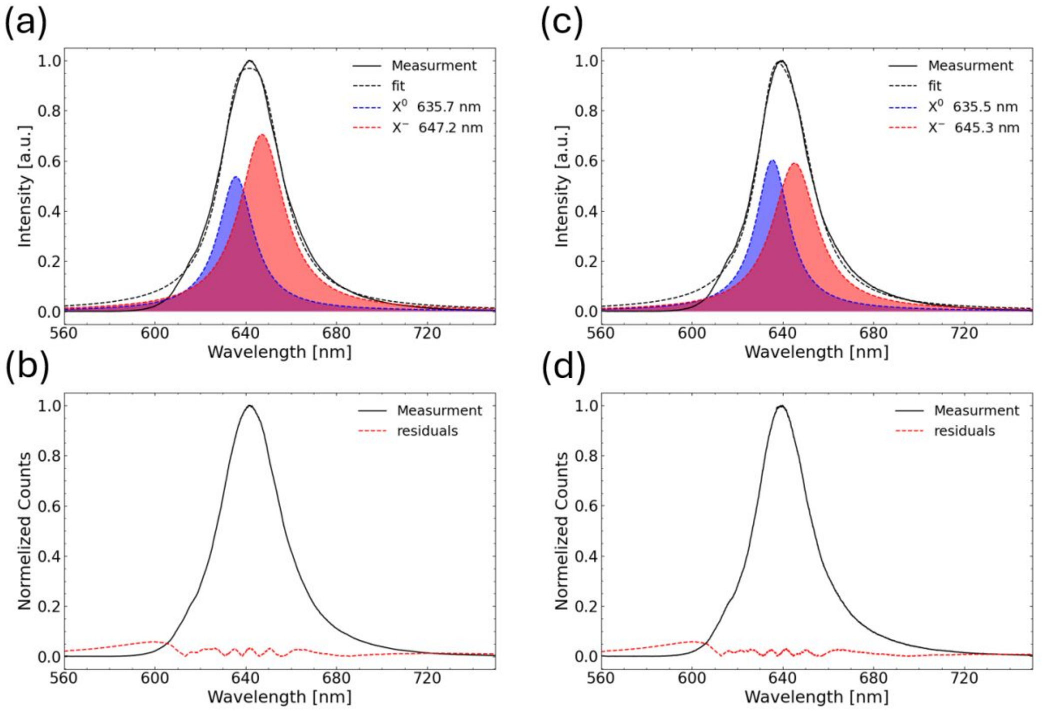

Figure S4. a) PL spectrum of monolayer WS_2 on a SiO_2 substrate fitted using a double Lorentzian decomposition. b) Corresponding residual error for the SiO_2 substrate. c) PL spectrum of monolayer WS_2 on an HfO_2 substrate with double Lorentzian fitting. d) Corresponding residual error for the HfO_2 substrate.

REFERENCES

(1) Zeng, H.; Liu, G. Bin; Dai, J.; Yan, Y.; Zhu, B.; He, R.; Xie, L.; Xu, S.; Chen, X.; Yao, W.; Cui, X. Optical Signature of Symmetry Variations and Spin-Valley Coupling in Atomically Thin Tungsten Dichalcogenides. *Sci Rep* **2013**, *3* (April). https://doi.org/10.1038/srep01608.

(2) Berkdemir, A.; Gutiérrez, H. R.; Botello-Méndez, A. R.; Perea-López, N.; Elías, A. L.; Chia, C. I.; Wang, B.; Crespi, V. H.; López-Urías, F.; Charlier, J. C.; Terrones, H.; Terrones, M. Identification of Individual and Few Layers of WS2 Using Raman Spectroscopy. *Sci Rep* **2013**, *3*, 1–8. https://doi.org/10.1038/srep01755.

(3) Molas, M. R.; Nogajewski, K.; Potemski, M.; Babiński, A. Raman Scattering Excitation Spectroscopy of Monolayer WS2. *Sci Rep* **2017**, *7* (1). https://doi.org/10.1038/s41598-017-05367-0.

(4) Wei, K.; Liu, Y.; Yang, H.; Cheng, X.; Jiang, T. Large Range Modification of Exciton Species in Monolayer WS_2. *Appl Opt* **2016**, *55* (23), 6251. https://doi.org/10.1364/ao.55.006251.

(5) Flatten, L. C.; He, Z.; Coles, D. M.; Trichet, A. A. P.; Powell, A. W.; Taylor, R. A.; Warner, J. H.; Smith, J. M. Room-Temperature Exciton-Polaritons with Two-Dimensional WS 2. *Sci Rep* **2016**, *6*. https://doi.org/10.1038/srep33134.

(6) Zhu, B.; Chen, X.; Cui, X. Exciton Binding Energy of Monolayer WS2. *Sci Rep* **2015**, *5*. https://doi.org/10.1038/srep09218.

(7) Shang, J.; Shen, X.; Cong, C.; Peimyoo, N.; Cao, B.; Eginligil, M.; Yu, T. Observation of Excitonic Fine Structure in a 2D Transition-Metal Dichalcogenide Semiconductor. *ACS Nano* **2015**, *9* (1), 647–655. https://doi.org/10.1021/nn5059908.

(8) Borghardt, S.; Tu, J. S.; Winkler, F.; Schubert, J.; Zander, W.; Leosson, K.; Kardynał, B. E. Engineering of Optical and Electronic Band Gaps in Transition Metal Dichalcogenide Monolayers through External Dielectric Screening. *Phys Rev Mater* **2017**, *1* (5), 1–8. https://doi.org/10.1103/PhysRevMaterials.1.054001.

(9) Peimyoo, N.; Wu, H. Y.; Escolar, J.; De Sanctis, A.; Prando, G.; Vollmer, F.; Withers, F.; Riis-Jensen, A. C.; Craciun, M. F.; Thygesen, K. S.; Russo, S. Engineering Dielectric Screening for Potential-Well Arrays of Excitons in 2D Materials. *ACS Appl Mater Interfaces* **2020**, *12* (49), 55134–55140. https://doi.org/10.1021/acsami.0c14696.
